# Comparison of the Efficacy and Safety Outcomes of Edoxaban in 8040 Women Versus 13 065 Men With Atrial Fibrillation in the ENGAGE AF-TIMI 48 Trial

**DOI:** 10.1161/CIRCULATIONAHA.120.052216

**Published:** 2021-02-16

**Authors:** Thomas A. Zelniker, Maddalena Ardissino, Felicita Andreotti, Michelle L. O’Donoghue, Ophelia Yin, Jeong-Gun Park, Sabina A. Murphy, Christian T. Ruff, Hans J. Lanz, Elliott M. Antman, Eugene Braunwald, Robert P. Giugliano, Piera Angelica Merlini

**Affiliations:** 1 Division of Cardiology, Medical University of Vienna, Austria (T.A.Z.).; 2 Department of Medicine, Imperial College, London, United Kingdom (M.A.).; 3 Fondazione Policlinico Universitario Gemelli IRCCS, Rome, Italy (F.A.).; 4 Institute of Cardiology, Catholic University Medical School, Rome, Italy (F.A.).; 5 TIMI Study Group, Cardiovascular Medicine, Brigham and Women’s Hospital, Harvard Medical School, Boston, MA (M.L.O., J.-G.P., S.A.M., C.T.R., E.M.A., E.B., R.P.G.).; 6 Daiichi Sankyo Inc, Basking Ridge, NJ (O.Y.).; 7 Daiichi Sankyo Europe GmbH, Munich, Germany (H.J.L.).; 8 Department of Cardiology, Niguarda Hospital, Milan, Italy (P.A.M.).

**Keywords:** anticoagulants, atrial fibrillation, factor Xa inhibitors, edoxaban, warfarin, women

## Abstract

**Background:**

Female sex is an independent risk factor for stroke and systemic embolic events in patients with atrial fibrillation. This study aimed to examine the efficacy and safety profile of edoxaban in women versus men.

**Methods:**

The ENGAGE AF-TIMI 48 trial (Effective Anticoagulation with Factor Xa Next Generation in Atrial Fibrillation-Thrombolysis in Myocardial Infarction 48) randomly assigned 21 105 patients (8040 women) with atrial fibrillation and CHADS_2_ score ≥2 either to a higher-dose edoxaban regimen, a lower-dose edoxaban regimen, or warfarin. The primary end points of the trial were the composite of stroke or systemic embolic events (efficacy), and International Society on Thrombosis and Haemostasis–defined major bleeding (safety).

**Results:**

In comparison with men, women were older, had lower body weight, were more likely to have hypertension and renal dysfunction, but less likely to smoke, drink alcohol, or have diabetes or coronary artery disease. Pretreatment endogenous factor Xa activity was significantly higher in women than in men (92.5% versus 86.1%, *P*<0.001). Treatment with edoxaban in women resulted in greater peak edoxaban concentration and inhibition of endogenous factor Xa in comparison with men, resulting in similar endogenous factor Xa activity between the sexes 2 to 4 hours after dose. Treatment with higher-dose edoxaban regimen (versus warfarin) resulted in similar reduction in the risk of stroke/systemic embolic events (women: hazard ratio [HR], 0.87 [0.69–1.11], men: HR, 0.87 [0.71–1.06]; *P*-interaction=0.97) and major bleeding (women: HR, 0.74 [0.59–0.92], men: HR, 0.84 [0.72–0.99]; *P*-interaction=0.34) in women and men. However, women assigned to higher-dose edoxaban regimen experienced greater reductions in hemorrhagic stroke (HR, 0.30 [95% CI, 0.15–0.59] versus HR, 0.70 [95% CI, 0.46–1.06]), intracranial bleeding (HR, 0.20 [95% CI, 0.10–0.39] versus HR, 0.63 [95% CI, 0.44–0.89]), and life-threatening or fatal bleeding (HR, 0.25 [95% CI, 0.15–0.42] versus HR, 0.72 [95% CI, 0.54–0.96]) than men (each *P*-interaction<0.05).

**Conclusions:**

Despite many differences in baseline characteristics between women and men and higher baseline endogenous factor Xa levels in women, the intensity of anticoagulation achieved with edoxaban between the sexes was similar. Treatment with higher-dose edoxaban regimen resulted in an even greater reduction in hemorrhagic stroke and several serious bleeding outcomes in women than in men, whereas the efficacy profile was similar between sexes.

## Abstract 2

Supplemental Digital Content is available in the text.

**Clinical Perspective**

**What Is New?**

- Female sex is an independent risk factor for stroke and systemic embolic events in patients with atrial fibrillation.
- Women had higher baseline endogenous factor Xa activity in comparison with men placing women at potential increased risk of thrombosis. Treatment with a higher-dose edoxaban regimen caused a greater reduction of anti-Xa activity in women than in men, resulting in similar intensity of achieved anticoagulation.
- The treatment effect of the higher-dose edoxaban regimen (versus warfarin) on the risk of stroke/systemic embolic events and major bleeding was similar in women and men.
- However, the higher-dose edoxaban regimen reduced the risk of several bleeding outcomes including hemorrhagic stroke to a greater extent in women than in men.

**What Are the Clinical Implications?**

- Despite very different baseline factors between men and women, the efficacy with edoxaban versus warfarin is preserved in women in comparison with men.
- The safety advantage of edoxaban in women is enhanced for multiple bleeding end points in comparison with men, suggesting that edoxaban is a particularly attractive option for the treatment of women with atrial fibrillation.

Over the past decades, sex-specific differences in clinical manifestations, therapeutic approaches, and prevention of cardiovascular diseases have been recognized. This prompted the need to increase the focus on sex-specific approaches to health care. Atrial fibrillation (AF) represents a major global burden and is associated with an increased risk of adverse cardiovascular events and death.^1,2^ The development of AF is known to be linked to several risk factors and comorbidities that vary between men and women, and several important differences in the epidemiology, pathobiology, symptoms, and prognosis of AF have been identified between the sexes.^3^ The age-adjusted incidence and prevalence of AF appear to be lower in women than in men, and there are sex-related differences in the clinical profile of AF: women are less likely to experience asymptomatic AF but more likely to present with atypical symptoms such as fatigue and report lower quality of life in comparison with men.^3–6^ Moreover, female sex has been shown to be an independent risk factor for stroke and systemic embolic events (SEE).^6–8^ This observation is reflected in the CHA_2_DS_2_-VASc score used to risk stratify patients with AF for the need of anticoagulation that assigns female sex 1 point as a risk factor for stroke.^9–11^

Edoxaban is a once-daily oral factor Xa (FXa) inhibitor that is noninferior to well-managed warfarin for the prevention of stroke or SEE in patients with AF and a CHADS_2_ score ≥2.^12^ In addition to preventing thromboembolic events, edoxaban significantly reduced the risk of bleeding and cardiovascular death in comparison with warfarin. However, higher pretreatment endogenous FXa activity (indicating a greater risk for thrombosis) has been reported in vitamin K antagonist–naive women in comparison with vitamin K antagonist–naive men.^13^ Despite these established sex-based differences in pretreatment FXa activity, potential sex-based differences in the efficacy and safety profile of edoxaban in comparison with warfarin have not yet been explored.

Therefore, the aims of this secondary analysis from the ENGAGE AF-TIMI 48 trial (Effective Anticoagulation with Factor Xa Next Generation in Atrial Fibrillation-Thrombolysis in Myocardial Infarction 48) were to compare (1) the risk of cardiovascular events, (2) the differences of pharmacokinetic and pharmacodynamic properties of edoxaban (using a direct measure of the functional activity of endogenous FX in plasma) and warfarin, and (3) the efficacy and safety of edoxaban and warfarin in women versus men with AF and a CHADS_2_ score ≥2.

## Methods

The data, analytic methods, and study materials will not be made available to other researchers for the purposes of reproducing the results or replicating the procedure. We encourage parties interested in collaboration and data sharing to contact the corresponding author directly for further discussions.

### Study Population and Procedures

The design and results of the ENGAGE AF-TIMI 48 trial have been described previously.^12,14^ In brief, this phase 3 multinational, double-blind, double-dummy, noninferiority trial enrolled 21 105 patients with AF. The eligibility criteria included AF documented by means of an electrocardiographic recording within 12 months of enrollment, and a CHADS_2_ score of ≥2. Patients were randomly assigned to a higher-dose edoxaban regimen (HDER; 60 mg once daily), a lower-dose edoxaban regimen (LDER; 30 mg once daily), or warfarin adjusted to an international normalized ratio (INR) of 2.0 to 3.0. The edoxaban dose was reduced by 50% in patients with any one of the following: (1) body weight of ≤60 kg, (2) estimated creatinine clearance of ≤50 mL/min, or (3) use of the potent P-glycoprotein inhibitors verapamil, quinidine, or dronedarone. Because only HDER is approved for clinical use in patients with AF, we focused on our analyses comparing HDER with warfarin.

### Pharmacokinetic and Pharmacodynamic Evaluation

Baseline endogenous FXa activity was assessed before administration of the first dose of study drug in 3114 patients. The peak-and-trough percentages of inhibition of endogenous FXa activity were measured at day 29 after randomization (median time from preceding dose, 20.0 hours; interquartile range (IQR), 15.4–24.3 hours) in 3224 and 3340 patients, respectively, at the TIMI Clinical Laboratory in Boston, Massachusetts, using a previously described validated assay.^13^ In addition, peak-and-trough extrinsic FXa and edoxaban plasma concentrations were assayed on day 29 in 3167and 2865 patients, respectively.^15^ As described previously, in contrast to anti-FXa assays used to measure in vitro the capacity of edoxaban to inhibit the activity of FXa that is added to plasma (therefore called exogenous anti-Xa), endogenous FXa assays measure the functional activity of endogenous FXa in plasma measured spectrophotometrically.^13^ Because FX in plasma is converted to FXa with Russell viper venom (in contrast with the preceding description where exogenous FXa is added), this then allows for measurement of the resultant FXa activity derived from the conversion of the patient’s own FX. Consequently, exogenous anti-FXa activity thus only serves as a surrogate for edoxaban plasma concentrations, while in contrast an endogenous FXa assay provides a direct measure of the functional activity of endogenous FX in plasma and therefore allows us to quantify the pharmacodynamic effect of edoxaban. Peak and trough plasma concentrations of edoxaban were measured in 10 345 and 6780 patients, respectively, by Quintiles Bioanalytical and ADME Laboratories (formerly Advion BioServices) using a validated turbo ion spray liquid chromatography mass spectrometry/mass spectrometry method with a lower limit of quantitation of 0.764 ng/mL.

Warfarin pharmacodynamics was assessed by INR measured at least monthly by using a point-of-care device. The time in the therapeutic range (TTR) was estimated by means of linear interpolation by individually calculating the TTR for each patient.^16^

### End Points

The same end points as previously described in the ENGAGE AF-TIMI 48 trial were used for the present subgroup analysis.^12,14^ The primary efficacy end point was the first occurrence of stroke or SEE. The key secondary efficacy end points were the composite of stroke, SEE, and cardiovascular mortality; all-cause mortality; and each component separately. The principal safety end point was major bleeding, as defined by the International Society on Thrombosis and Haemostasis.^17^ Prespecified secondary bleeding end points, including fatal or life-threatening bleeding, intracranial hemorrhage, major or clinically relevant nonmajor bleeding, and all bleeding were also analyzed. The previously defined primary net clinical outcome included all-cause death, stroke, SEE, and major bleeding.

All elements of the efficacy, safety, and net clinical end points were adjudicated by a Clinical Events Committee that was unaware of randomized treatment allocation.

### Statistical Analysis

Baseline characteristics stratified by sex are summarized using means (and standard deviation) or medians (and quartiles) as appropriate. Outcome event rates are expressed as the total number of events (rate per 100 patient-years of follow-up). The association between sex and outcomes was calculated within the warfarin arm by using Cox proportional hazard regression models adjusted for age, body mass index, race, smoking, baseline serum creatinine, previous stroke or transient ischemic attack, diabetes, heart failure, increased risk of falling, neuropsychiatric disease, hypertension, coronary artery disease, dyslipidemia, history of hepatic disease, history of extracranial hemorrhage, alcohol intake, medication predisposing to bleeding, and pattern of AF.

Cox regression models with interaction testing were applied to test for effect modification by sex. The proportional hazards assumption was tested and verified for each of the major study end points (primary efficacy, primary safety, primary net outcome) and sex (men versus women) by using Schoenfeld residuals. In addition, sensitivity analyses were performed using multivariable Cox regression models that were adjusted for differences in baseline characteristics including age, body mass index, race, smoking, history of stroke or transient ischemic attack, diabetes, heart failure, hypertension, coronary artery disease, dyslipidemia, peripheral artery disease, history of hepatic disease, history of extracranial hemorrhage, and pattern of AF.

As described previously,^13^ the pharmacokinetics and pharmacodynamics of edoxaban were assessed using nonlinear mixed-effects modeling, with stepwise forward-addition and backward-elimination processes for covariate election, including age, sex, race, body weight, smoking, history of stroke or transient ischemic attack, diabetes, heart failure, hypertension, coronary artery disease, peripheral artery disease, aspirin use, platelet count, and serum creatinine entered as candidate predictors. The relationship between baseline characteristics and FXa activity was assessed by using linear regression models after multivariable adjustment using a backward-forward stepwise model selection including the same candidate predictors as used for the pharmacokinetic and pharmacodynamic modeling of edoxaban. All reported *P* values are 2-sided and *P*<0.05 was considered to signify nominal statistical significance. No statistical adjustments were made for multiple comparisons.

The protocol and amendments were approved by the Ethics Committee at each participating center. All patients provided their written informed consent. The TIMI Study Group maintained an independent copy of the trial database and conducted the current analysis. Analyses were performed using STATA/SE version 12.1 (Stata Corp) and SAS 9.4.

## Results

### Baseline Characteristics

The baseline characteristics of the 21 105 patients are summarized by sex in Table 1. Overall, 8040 women (38.1%) were included in the trial. In comparison with men, their median weight was 12 kg less but their body mass index was slightly higher (median 28.9 versus 28.5 kg/m^2^). Moreover, in this trial population, women had a median age 3 years greater, were more likely to have hypertension, paroxysmal AF (as opposed to persistent or permanent AF), valvular heart disease, and abnormal renal function, whereas they were less likely to smoke, drink alcohol, have diabetes, carotid artery disease, or a previous myocardial infarction, or have undergone coronary revascularization.

**Table 1.**
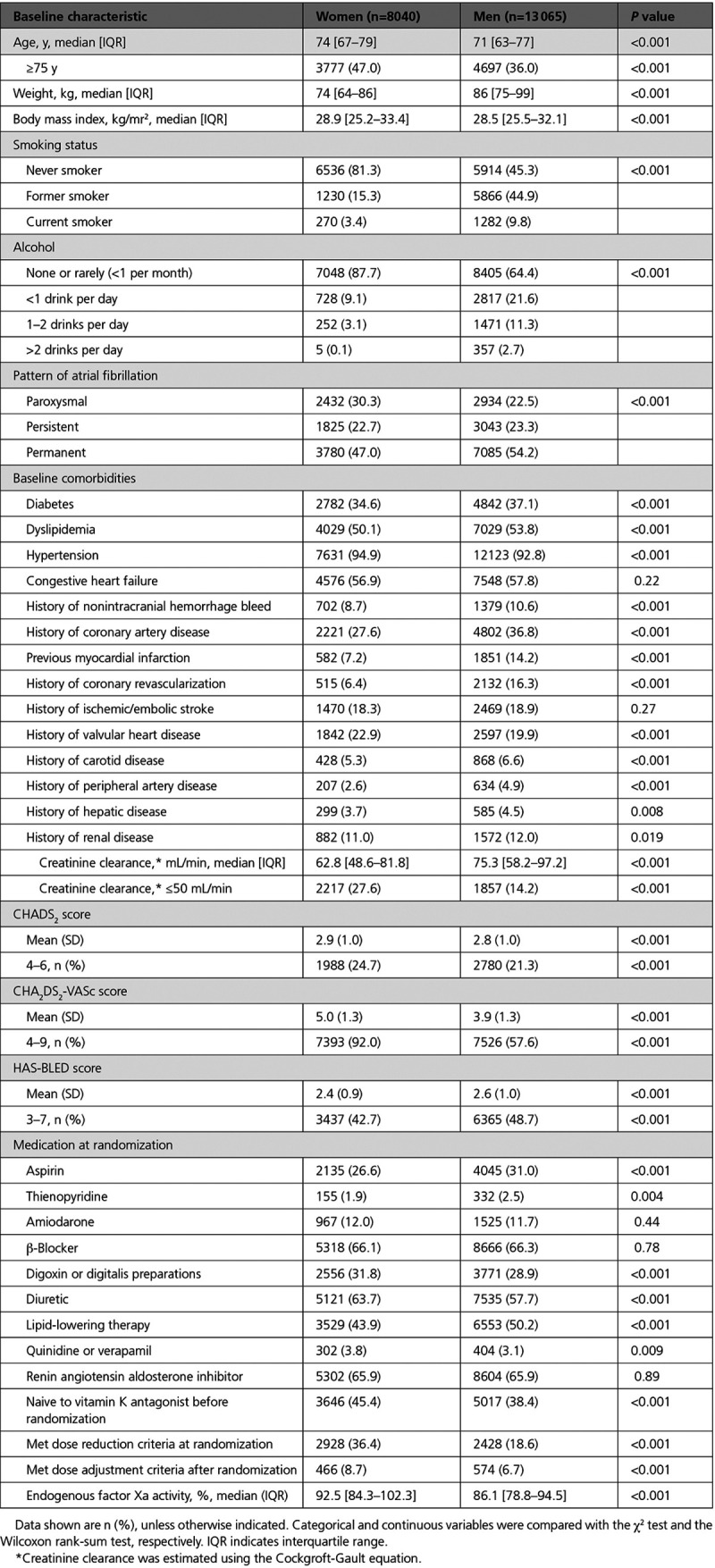
Baseline Characteristics of Patients According to Sex

Women were less likely to be receiving anticoagulant or antiplatelet therapy at the time of randomization, but they received digoxin more frequently. In comparison with men, a higher proportion of women had a CHA_2_DS_2_-VASc score ≥4 (92.0% versus 57.6%; *P*<0.0001), but a smaller proportion of women had high bleeding risk (HAS-BLED ≥3; 42.7% versus 48.7%; *P*<0.0001). The protocol-mandated 50% dose reduction of edoxaban was made in 36.4% of women versus 18.6% of men (*P*<0.0001) at randomization. An additional 8.7% of women versus 6.7% of men (*P*<0.001) met dose adjustment criteria during follow-up.

### Outcomes in the Warfarin Group According to Sex

Within the warfarin treatment arm, women had a similar risk of stroke or SEE after multivariable adjustment (annualized rate 2.00% versus 1.67%; adjusted hazard ratio (HR), 1.21 [95% CI, 0.94–1.56]; *P*=0.14) in comparison with men (Table 2). There were no significant differences in the secondary efficacy outcomes between women and men assigned to warfarin (Table 2). Moreover, the rates of major bleeding in the warfarin group were similar in women and men (3.35% versus 3.47% per year; adjusted HR, 0.90 [95% CI, 0.72–1.12]; *P*=0.34), but women had statistically significant higher rates of major or clinically relevant nonmajor bleeding (10.63% versus 9.87% per year; adjusted HR, 1.16 [95% CI, 1.02–1.32]; *P*=0.021) and overt bleeding episodes (17.14% versus 15.97% per year; adjusted HR, 1.14 [95% CI, 1.02–1.26]; *P*=0.017).

**Table 2.**
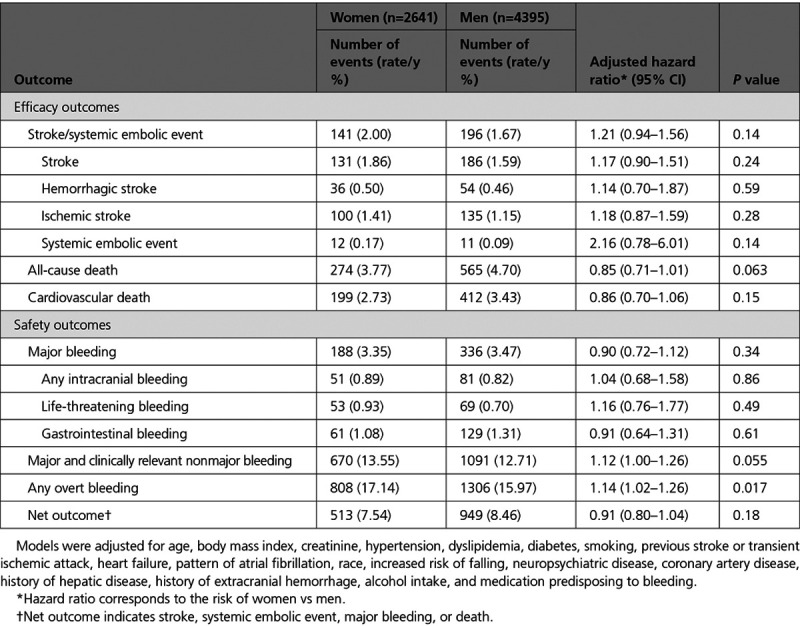
Efficacy and Safety in the Warfarin Arm According to Sex

### Pharmacokinetics and Pharmacodynamics of Edoxaban and Warfarin in Men Versus Women

The baseline (ie, pretreatment) endogenous FXa activity was significantly higher in warfarin-naive women in comparison with men (women: 92.5%, IQR 84.3–102.3 versus men 86.1%, IQR 78.8–94.5; *P*<0.001; Figure I in the Data Supplement). Female sex remained significantly associated with higher endogenous FXa activity (coefficient estimate, 4.73 [standard error±1.23]; *P*=0.0001) after multivariable adjustment using a stepwise model selection.

**Figure 1.**
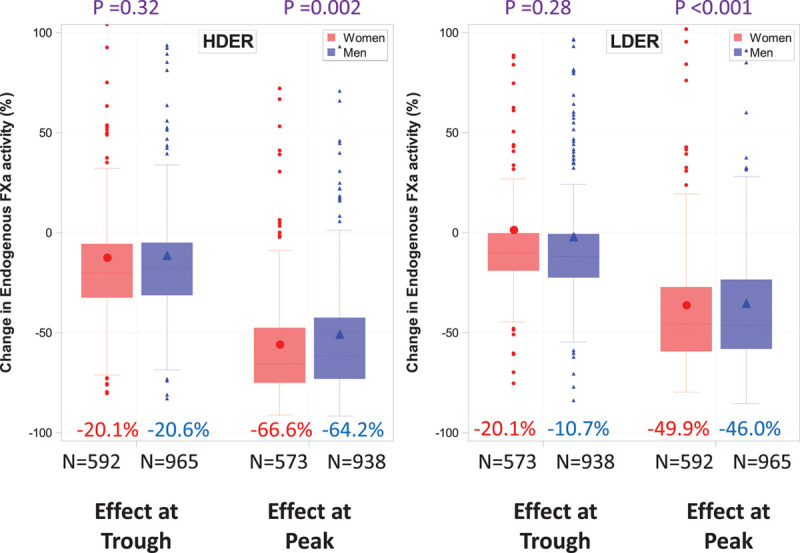
**Change in endogenous FXa activity at 29 days.** Values are expressed as percent inhibition (median) of baseline FXa at peak and trough with HDER and LDER. The difference in the change in endogenous FXa between men and women was tested using the nonparametric Wilcoxon rank-sum test. FXa indicates factor Xa; HDER, higher-dose edoxaban regimen; and LDER, lower-dose edoxaban regimen.

**Figure 2.**
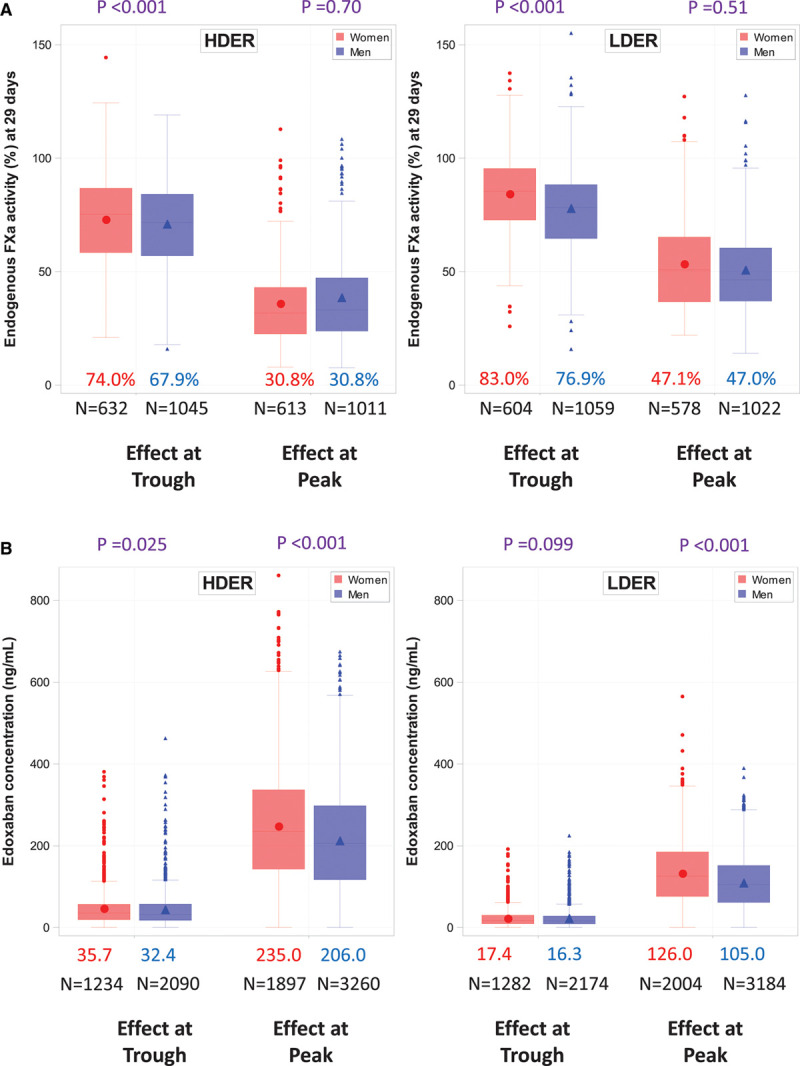
**Peak-and-trough endogenous FXa activity and edoxaban concentrations with HDER and LDER in men and women at 29 days.** **A**, Peak-and-trough endogenous FXa activity. **B**, Edoxaban concentrations. Data shown are medians. The difference between men and women in the change in peak-and-trough endogenous FXa activity and edoxaban concentrations was tested in each case using the nonparametric Wilcoxon rank-sum test. FXa indicates factor Xa; HDER, higher dose edoxaban regimen; and LDER, lower-dose edoxaban regimen.

Treatment with both HDER and LDER resulted in greater inhibition of the baseline endogenous FXa at peak effect in women in comparison with men (Figure 1), leading to similar measured levels of endogenous FXa activity between the 2 sexes 2 to 4 hours after dose on day 29 (Figure 2A). However, among patients who were assigned to HDER and who met criteria for dose reduction, women had even greater inhibition of baseline endogenous FXa activity than men at peak effect (endogenous FXa activity in women: 34.4%, IQR 27.5–45.9 versus men: 38.8%, IQR 30.6–51.6; *P*=0.013; Figure II in the Data Supplement). There was no significant difference in the change of endogenous FXa inhibition from baseline to the trough effect (ie, just before the next dose) between women and men (Figures 1 and 2A).

HDER resulted in significantly higher peak-and-trough edoxaban concentrations on day 29 in women than in men (peak: 235.0 versus 206.0 ng/mL, *P*<0.001; trough: 35.7 versus 32.4 ng/mL, *P*=0.025; Figure 2B). Similarly, women assigned to LDER had higher peak edoxaban levels and tended to have higher trough edoxaban levels than men (peak: 126.0 versus 105.0 ng/mL, *P*<0.001; trough: 17.4 versus 16.3 ng/mL, *P*=0.099; Figure 2B, Figure III in the Data Supplement). Similar patterns were observed for exogenous FXa concentrations (Figures IV and V in the Data Supplement ).

Among patients randomly assigned to warfarin, the median TTR was lower in women than in men (median TTR 67.1%, IQR 54.6%–76.1% versus 69.3%, IQR 57.9%–78.2%; *P*<0.001). Specifically, warfarin-treated women were more likely to have subtherapeutic anticoagulation levels than men (median time of INR <2.0; 18.7%, IQR 11.6%–30.2% versus 17.2%, IQR 10.6%–27.0%; *P*<0.001). There was no significant difference in the median proportion of time with INR >3.0 between women and men assigned to warfarin (women: 11.1%, IQR 5.9%–16.8% versus men: 10.7%, IQR 5.8%–16.5%; *P*=0.101).

### Efficacy and Safety of Edoxaban Versus Warfarin in Men and Women

**Figure 3.**
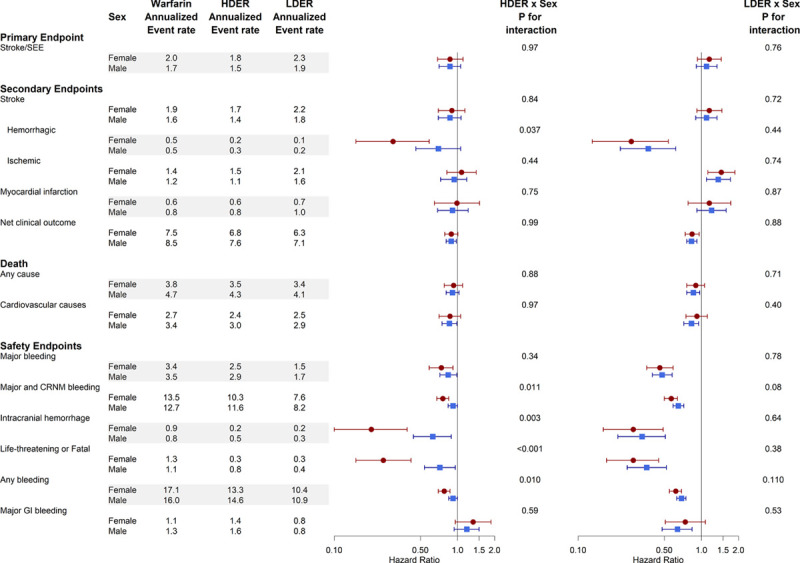
**Efficacy and safety of edoxaban vs warfarin stratified by sex.** The *x* axis of the forest plot represents the hazard ratio. CRNM indicates clinically relevant nonmajor; HR, hazard ratio; GI, gastrointestinal; HDER, higher-dose edoxaban regimen; LDER, lower dose edoxaban regimen; and SEE, systemic embolic events.

The treatment effect of edoxaban versus warfarin in men and women has been reported previously for the primary efficacy and safety outcomes.^12^ The effect of HDER on the risk of the primary efficacy end point of stroke/SEE was similar in women (HR, 0.87 [95% CI, 0.69–1.11]) and men (HR, 0.87 [95% CI, 0.71–1.06], *P*-interaction=0.97; Figure 3). Similarly, the point estimates of the HRs for the primary safety end point major bleeding did not differ between women (HR, 0.74 [95% CI, 0.59–0.92]) and men (HR, 0.84 [95% CI, 0.72–0.99]; *P*-interaction=0.34; Figure 3).

However, the reduction in hemorrhagic stroke obtained with HDER in comparison with warfarin was significantly greater in women than in men (women: HR, 0.30 [95% CI, 0.15–0.59] versus men: HR, 0.70 [95% CI, 0.46–1.06]; *P*-interaction=0.037). No significant interactions in other efficacy outcomes were observed for the comparison of the HDER versus warfarin. However, among the secondary safety end points, women assigned to HDER experienced greater reductions in life-threatening or fatal bleeding (HR, 0.23 [95% CI, 0.13–0.39] versus HR, 0.71 [95% CI, 0.53–0.96]; *P*-interaction<0.001), intracranial bleeding (HR, 0.20 [95% CI, 0.10–0.39] versus HR, 0.63 [95% CI, 0.44–0.89]; *P*-interaction=0.003), major or clinically relevant nonmajor bleeding (HR, 0.76 [95% CI, 0.68–0.85] versus HR, 0.92 [95% CI, 0.84–1.00]; *P*-interaction=0.011) and any bleeding episodes (HR, 0.78 [95% CI, 0.70–0.87] versus HR, 0.92 [95% CI, 0.85–1.00]; *P*-interaction=0.010) than men. The increase in major gastrointestinal bleeding with HDER in comparison with warfarin was similar in women and men (women: HR, 1.34 [95% CI, 0.96–1.87], men: HR, 1.19 [95% CI, 0.94–1.50]; *P*-interaction=0.59). Sensitivity analyses using multivariable adjusted Cox regression models yielded nearly identical point estimates (but more precise confidence intervals) and *P* values for interaction (Figure VI in the Data Supplement). Moreover, the differences in the secondary safety end points between women and men treated with HDER (versus warfarin) remained apparent when patients were stratified by criteria for dose reduction (Figures VII and VIII in the Data Supplement).

### Effect of Edoxaban Versus Warfarin in Women by Major Subgroups

The numeric reduction in stroke/SEE with HDER (versus warfarin) was consistent in women regardless of age at baseline (<65 years: HR, 0.83 [95% CI, 0.39–1.78]; 65–75 years: HR, 0.83 [95% CI, 0.54–1.29]; *P*-interaction=1.00; ≥75 years: HR, 0.90 [95% CI, 0.66–1.23]; *P*-interaction=0.86) or CHADS_2_ score at baseline (CHADS_2_ score ≤3: HR, 0.93 [95% CI, 0.68–1.26], CHADS_2_ score >3: HR, 0.79 [95% CI, 0.54–1.17]; *P*-interaction=0.53; Figure IX in the Data Supplement).

In women, HDER (versus warfarin) significantly reduced the risk of major bleeding irrespective of baseline age (<65 years: HR, 0.93 [95% CI, 0.49–1.78]; 65–75 years: HR, 0.77 [95% CI, 0.52–1.15]; *P*-interaction=0.64; ≥75 years: HR, 0.68 [95% CI, 0.51–0.92]; *P*-interaction=0.40) or CHADS_2_ score at baseline (CHADS_2_ score ≤3: HR, 0.73 [95% CI, 0.56–0.95], CHADS_2_ score >3: HR, 0.75 [95% CI, 0.50–1.14]; *P*-interaction=0.89; Figure X in the Data Supplement).

Moreover, the treatment effect of HDER on stroke/SEE and major bleeding in women was not modified by a history of heart failure, hypertension, previous myocardial infarction, aspirin use, and those who met versus who did not meet dose reduction criteria (all *P*-interaction>0.05; Figures IX and X in the Data Supplement).

### Results With the LDER

The edoxaban concentration and exogenous FXa activity were ≈50% lower with LDER (30 mg, dose reduced to 15 mg) in comparison with the approved HDER (60/30 mg), and did not differ between women and men (Figure 2B, Figures III–V in the Data Supplement). This translated into more modest reductions in the inhibition of baseline endogenous FXa activity on day 29 with LDER (peak 46%–50%, trough 11%; Figure 1) irrespective of sex. Ischemic stroke was significantly increased, whereas all bleeding end points, mortality, and the net outcome were decreased with LDER versus warfarin, to a similar degree in women and men (each *P*-interaction>0.05; Figure 3 and Figures VI–VIII in the Data Supplement). Results with LDER versus warfarin in subgroups of women were consistent with the overall findings (Figure IX in the Data Supplement).

## Discussion

The principal findings of this secondary analysis from ENGAGE AF-TIMI 48 that included 8040 women with AF at moderate to high risk of stroke were that the magnitude of reduction in multiple serious bleeding end points with HDER versus warfarin, including intracranial hemorrhage, fatal or life-threatening bleeding, and the composite of clinically relevant major or nonmajor bleeding, were greater in women than in men. Stroke or SEE, cardiovascular death, and the net outcome were reduced to a similar degree in both women and men.

Similar to previous studies,^6,8^ higher rates of stroke but lower event rates of cardiovascular death occurred in women in comparison with men, yet these differences did not meet statistical significance after multivariable adjustment. However, women had a significantly higher risk of clinically relevant nonmajor and any overt bleeding than men after multivariable adjustment.

This study gives insight into the sex-related pharmacokinetics and pharmacodynamics of edoxaban and warfarin. Throughout the trial, the median TTR was high, but 2% lower in women, thus indicating slightly better control of the INR in men. This is particularly relevant because it was observed in participants of a clinical trial that ensured frequent and centralized INR monitoring and was characterized by a substantially higher TTR than that reported in routine clinical practice.^6,11^ The present data, therefore, highlight challenges of warfarin management in women. With respect to FXa, women start at a higher level of activity (ie, higher thrombotic potential) than men before anticoagulation. This finding was present even after multivariable adjustment for the many differences in baseline characteristics including weight and age present between the sexes and support the 1 point given to female sex in the CHA_2_DS_2_-VASc score.^11,18^ The observation that sex is an independent predictor of FXa activity even after accounting for weight and age is supported by previous secondary analyses from this trial that did not observe important pharmacokinetic or pharmacodynamic differences dependent on body mass index, or extremes of body weight.^13,19,20^ Despite a higher baseline endogenous FXa level in women, the percentage of inhibition of the baseline endogenous FXa activity with HDER was greater in women, thus resulting in similar levels of FXa activity at peak effect between the sexes. These observations were consistent in men and women who did or did not meet criteria for edoxaban dose reduction at randomization.

The safety profile of HDER in comparison with warfarin was even more favorable in women than in men, even after accounting for baseline differences between the sexes in a sensitivity analysis. This could be related, at least in part, to the higher proportion of women who qualified for dose reductions: 36% versus 19% at the time of randomization, and 8.7% versus 6.7% during follow-up. It has been recognized that patients who meet dose-adjustment criteria are at higher risk of both ischemic and bleeding events than those who do not meet these criteria.^15^ It is noteworthy that an analysis of the causes of death and their relationships to bleeding in the ENGAGE AF-TIMI 48 trial found that more than half of the reduction in deaths in the edoxaban-treated patients could be attributed to a reduction in major bleeding.^21^ It is therefore possible that the dose reduction criteria selected in this trial were particularly favorable for women, and a sweet spot balancing antiischemic efficacy and the risk of bleeding was found. However, further in-depth analyses restricting patients who did and did not meet criteria for dose reduction indicate a consistently more favorable safety profile in women than in men. It is unlikely that the lower TTR in women had contributed to the observed treatment heterogeneity for safety events because the event rates in the warfarin treatment arm were similar in both sexes (or even slightly higher in women), and women were significantly more likely to have a subtherapeutic INR than men.

In contrast, secondary analyses from the other direct oral anticoagulant versus warfarin trials did not report differences in the safety (or efficacy) profile of apixaban, rivaroxaban, or dabigatran between women and men.^22–26^ An analysis of 6418 women in the ARISTOTLE trial (Apixaban for the Prevention of Stroke in Subjects With Atrial Fibrillation) showed that both efficacy and safety (including intracranial bleeding) of apixaban were consistent in both sexes.^22^ In ARISTOTLE, a 50% dose reduction was required for patients with at least 2 of the following characteristics: age ≥80 years, weight ≤60 kg, and serum creatinine concentration ≥1.5 mg/mL. Only 4.7% of the study population had the dose reduced, and dose reductions were performed only at the time of randomization.

In the ROCKET AF trial (An Efficacy and Safety Study of Rivaroxaban With Warfarin for the Prevention of Stroke and Non-Central Nervous System Systemic Embolism in Patients With Non-Valvular Atrial Fibrillation), there was no interaction between sex and the treatment effect of rivaroxaban on stroke/SEE, but women assigned to rivaroxaban had significantly fewer bleeding events in comparison with men.^26^ Of note, the dose of rivaroxaban was reduced by only 25% (from 20 to 15 mg) and only in the patients whose creatinine clearance was 30 to 50 mL/min at randomization; there was no dose adjustment for body weight or drug-drug interactions, or dose adjustments after randomization.

The RE-LY trial (Randomized Evaluation of Long-Term Anticoagulation Therapy) tested 2 dabigatran doses and warfarin without using predefined dose adjustments in special populations. The treatment effect was similar in women and men in the main trial. An exposure-response analysis of the RE-LY trial found that plasma concentrations of dabigatran were ≈30% higher in women than in men,^27,28^ larger than the sex-based differences in edoxaban concentrations in the present analysis. The relatively higher plasma dabigatran concentration in women might explain why neither dabigatran dose reduced bleeding rates in women.

### Strengths and Limitations

The data set of the ENGAGE AF-TIMI 48 trial represents the largest patient cohort of patients with AF participating in a randomized trial (n=21 105) and includes the largest number of women (n=8040) who were followed up for an average of nearly 3 years. Pharmacokinetic and pharmacodynamic measurements of edoxaban were obtained at peak and trough, which helps interpret the clinical findings.^13^ Moreover, the median TTR was 68.4% among patients treated with warfarin, which is higher than that reported in previous direct oral anticoagulant versus warfarin trials^24–26^ or in a US assessment of 138 319 Americans with AF (median TTR 57.5%).^29^

Despite the large sample size and the well-characterized patient population, several limitations need to be considered. Because pharmacokinetic and pharmacodynamic measurements were performed only in a subset of patients, potential selection bias is possible, although the number of patients was large and the baseline characteristics between patients with and without pharmacokinetic and pharmacodynamic data were similar.^15^ Although higher FXa activity in women (versus men) at baseline measured in this trial is consistent with the observation of higher thromboembolic risk in women who are not anticoagulated (and hence support 1 point for women in CHA_2_DS_2_-VASc score), we did not perform a comprehensive assessment of coagulant factors. Furthermore, because all patients in ENGAGE AF-TIMI 48 were treated with an anticoagulant, we could not test the hypothesis of a link between baseline FXa levels and ischemic stroke/SEE rates after randomization. Moreover, differences in the safety profile of HDER between men and women were most notable in secondary end points, for which the study was not powered. Although the observed differences in FXa activity between men and women might offer a pathobiological explanation, the impact of age and body weight might not be eliminated completely despite adjustment for these variables. In addition to the known limitations of subgroup analyses, this randomized, controlled trial included patients who met prespecified entry criteria and thus might not be generalizable to all patients with AF. However, the clinical characteristics of the patients in the ENGAGE AF-TIMI 48 trial are similar to those described in large registries and large observational studies.^2,4,6–8^ Because the exploratory and hypothesis-generating nature of this analysis, no adjustments for multiple testing were performed.

### Conclusions

Although women at baseline had a higher baseline level of FXa activity placing women at a potentially increased risk of thrombosis relative to men, treatment with HDER reduced the endogenous anti-Xa activity to a greater extent in women than in men. HDER (versus warfarin) reduced the risk of several bleeding outcomes to an even greater extent in women than in men. The efficacy profile of HDER relative to warfarin, including significant reductions in cardiovascular mortality and the net outcome, with similar rates of ischemic events, was consistent irrespective of sex. The overall balance of favorable efficacy and an enhanced safety profile of edoxaban in women suggests that edoxaban is a particularly attractive option for the treatment of women with AF and CHADS_2_ ≥2.

## Sources of Funding

The ENGAGE AF–TIMI 48 trial (Effective Anticoagulation with Factor Xa Next Generation in Atrial Fibrillation-Thrombolysis in Myocardial Infarction Study 48) was supported by a research grant from Daiichi-Sankyo to the Brigham and Women’s Hospital. No additional financial support was received for this study.

## Disclosures

Dr Zelniker reports research grants from German Research Foundation (Deutsche Forschungsgemeinschaft) and Austrian Science Fund (FWF), and speaking and consulting fees from AstraZeneca, personal fees from Boehringer Ingelheim, as well. Dr Andreotti reports receiving speaking and consulting fees from Amgen, Bayer, Boehringer Ingelheim, Bristol-Myers Squibb–Pfizer, and Daiichi Sankyo. Dr O’Donoghue reports: previous grants from in past 3 years: Eisai, AstraZeneca, GlaxoSmithKline, Merck, Amgen, and Janssen. Current grants from Medicines Company/Novartis, Amgen, Medimmune. Consulting: Novartis, Janssen, CRICO, AstraZeneca, Amgen, Honoraria: Medscape Cardiology. Dr Yin is an employee of Daiichi Sankyo Inc. Dr Park is a member of the TIMI Study Group, which has received institutional research grant support through Brigham and Women’s Hospital from Abbott, Amgen, Anthos Therapeutics, AstraZeneca, Daiichi-Sankyo, Eisai, Intarcia, MedImmune, Merck, Novartis, Pfizer, Regeneron Pharmaceuticals, Inc, Roche, The Medicines Company, and Zora Biosciences. S.A. Murphy is a member of the TIMI Study Group, which has received institutional research grant support through Brigham and Women’s Hospital from Abbott, Amgen, Anthos Therapeutics, AstraZeneca, Daiichi-Sankyo, Eisai, Intarcia, MedImmune, Merck, Novartis, Pfizer, Regeneron Pharmaceuticals, Inc., Roche, The Medicines Company, and Zora Biosciences. Dr Ruff reports research grant through institution: Anthos, Boehringer Ingelheim, Daiichi Sankyo, AstraZeneca, and National Institutes of Health; honoraria for scientific advisory boards and consulting: Anthos, Bayer, Bristol Myers Squibb, Boehringer Ingelheim, Daiichi Sankyo, Janssen, Pfizer, and Portola. Dr Ruff is a member of the TIMI Study Group, which has received institutional research grant support through Brigham and Women’s Hospital from Abbott, Amgen, Anthos Therapeutics, AstraZeneca, Daiichi-Sankyo, Eisai, Intarcia, MedImmune, Merck, Novartis, Pfizer, Regeneron Pharmaceuticals, Inc, Roche, The Medicines Company, and Zora Biosciences. Dr Lanz is an employee of Daiichi Sankyo Europe GmbH. Dr Antman reports receiving grant support through his institution from Daiichi Sankyo. Dr Braunwald reports research grants (through the Brigham and Women’s Hospital) from Astra Zeneca, Daiichi-Sankyo, Merck, and Novartis, and consultancies with Amgen, Boehringer-Ingelheim/Lilly, Cardurion, IMMEDIATE, MyoKardia, NovoNordisk, and Verve. Dr Giugliano reports clinical trials/research support: Amgen, Anthos Therapeutics, Daiichi Sankyo; honoraria for CME lectures: Amgen, Daiichi Sankyo, Merck, SAJA Pharmaceuticals, and Servier; Consultant: Amarin, American College of Cardiology, Amgen, Astra Zeneca, Boehringer-Ingelheim, Bristol-Myers-Squibb, CryoLife, CVS Caremark, Daiichi Sankyo, Eli Lilly and Company, Esperion, Gilead, GlaxoSmithKline, Janssen, Lexicon, Merck, Pfizer, and Samsung. Dr Merlini reports consulting fees from Amgen and Daiichi Sankyo. Dr Ardissino reports no conflicts.

## Supplemental Materials

Data Supplement Figures I–X

## Supplementary Material


