# Supplementary material for: Comparison of the Efficacy and Safety Outcomes of Edoxaban in 8040 Women Versus 13 065 Men With Atrial Fibrillation in the ENGAGE AF-TIMI 48 Trial
Source: Circulation. 2021 Feb 16;143(7):673–84. doi: 10.1161/CIRCULATIONAHA.120.052216 (PMC7884089; doi:10.1161/CIRCULATIONAHA.120.052216)

Appendix

Supplemental Figure I: Baseline endogenous Factor Xa activity according to dose-reduction criteria in men and women.

The difference between men and women was tested using the non-parametric Wilcoxon rank-sum test. DR = dose reduction

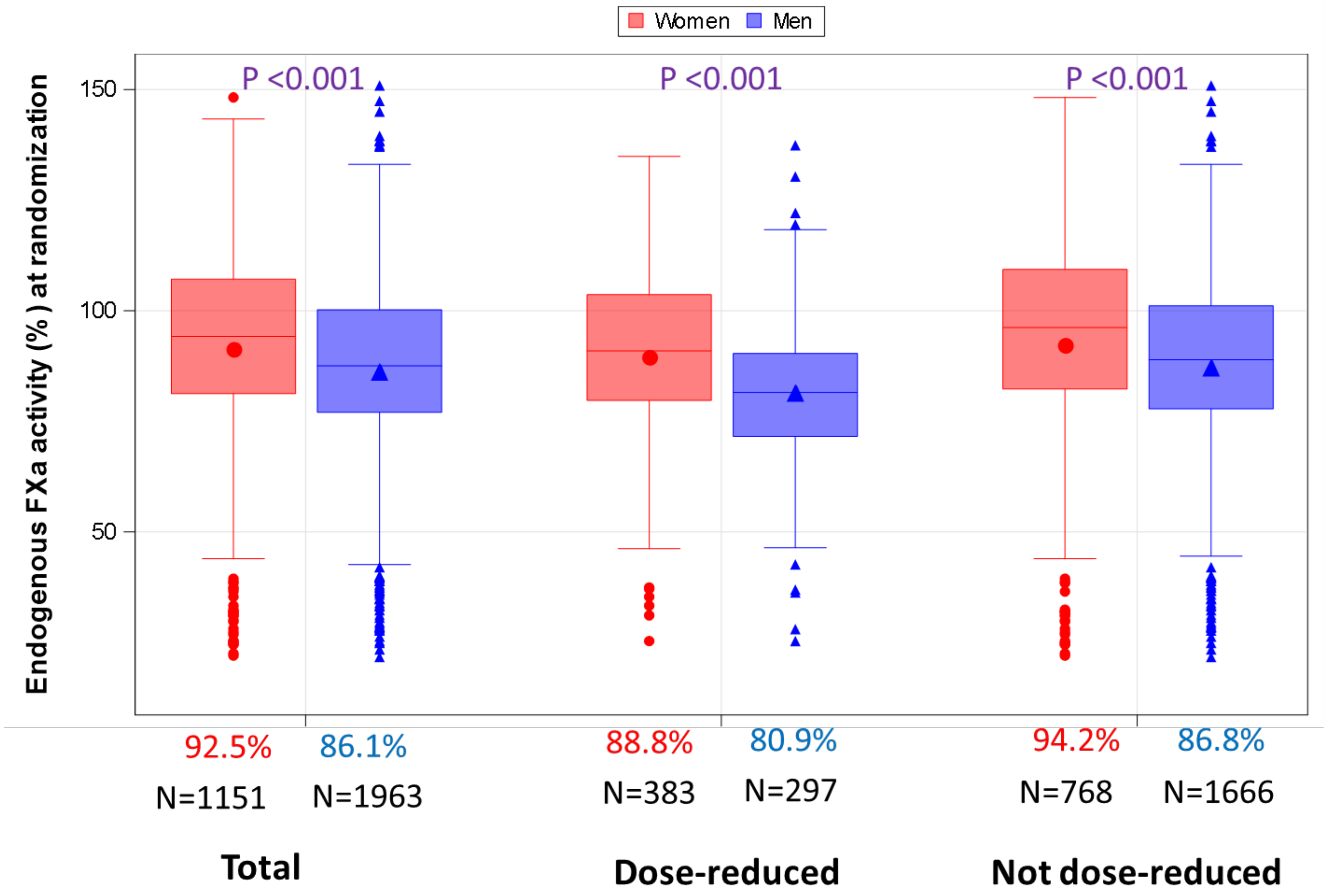

Supplemental Figure II: Endogenous Factor Xa activity with higher-dose (HDER) and lower-dose edoxaban regimen (LDER) according to dose-reduction criteria.

The difference between men and women was tested using the non-parametric Wilcoxon rank-sum test.

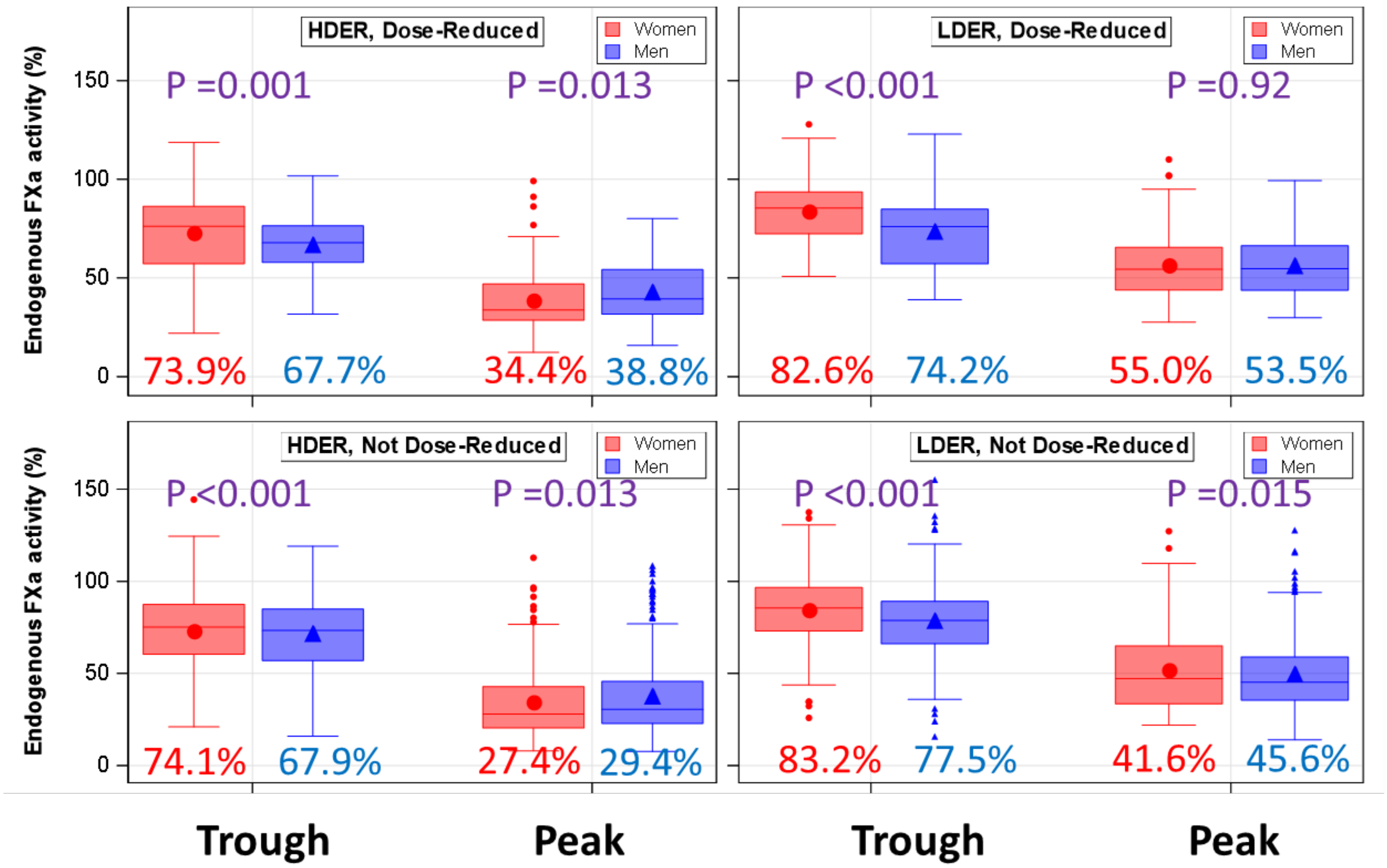

Supplemental Figure III: Edoxaban concentrations with higher-dose (HDER) and lower-dose edoxaban regimen (LDER) according to dose-reduction (DR) criteria.

The difference between men and women was tested using the non-parametric Wilcoxon rank-sum test.

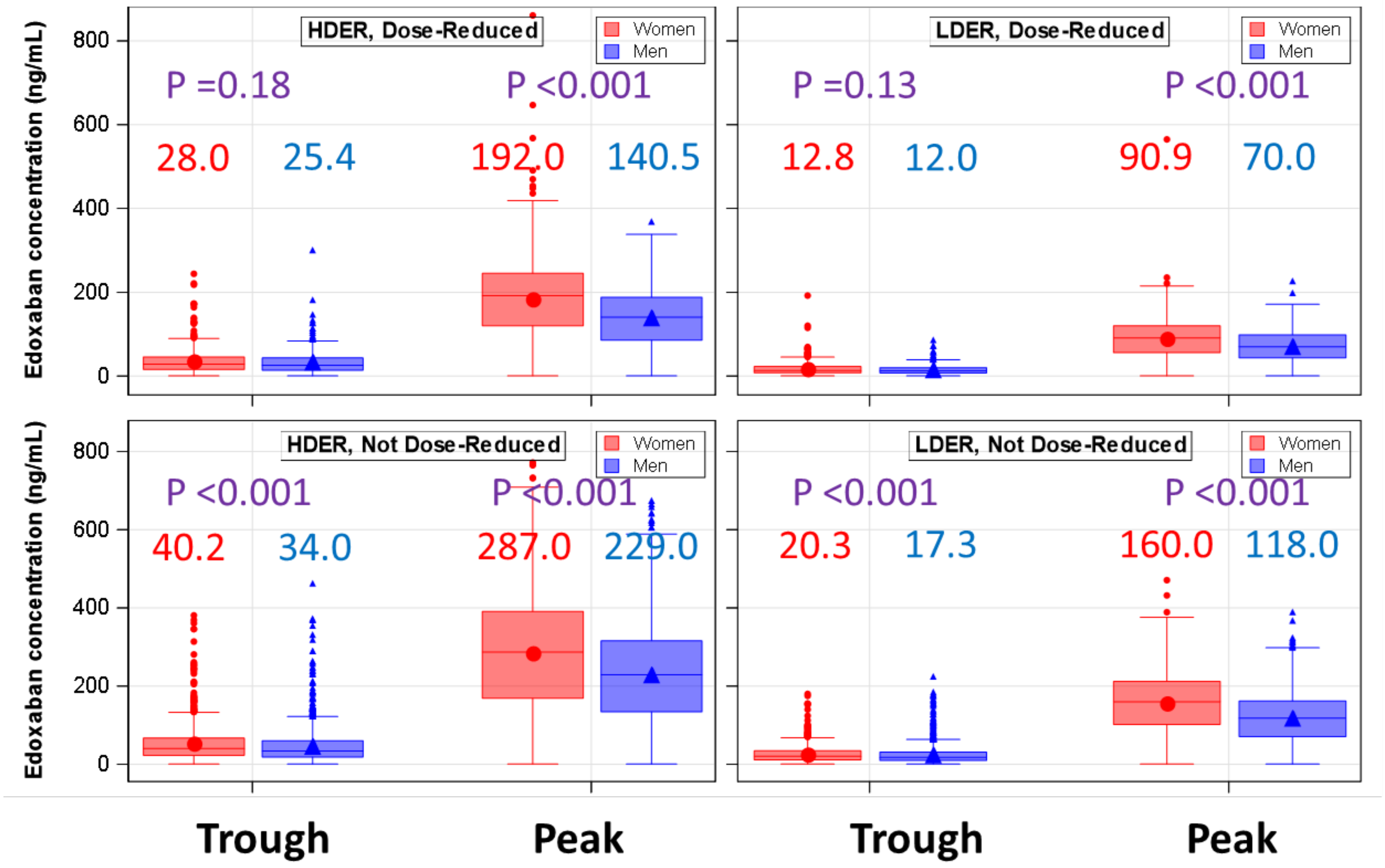

Supplemental Figure IV: Exogenous Factor Xa Concentrations with higher-dose (HDER) and lower-dose edoxaban (LDER) regimen. The difference between men and women was tested using the non-parametric Wilcoxon rank-sum test.

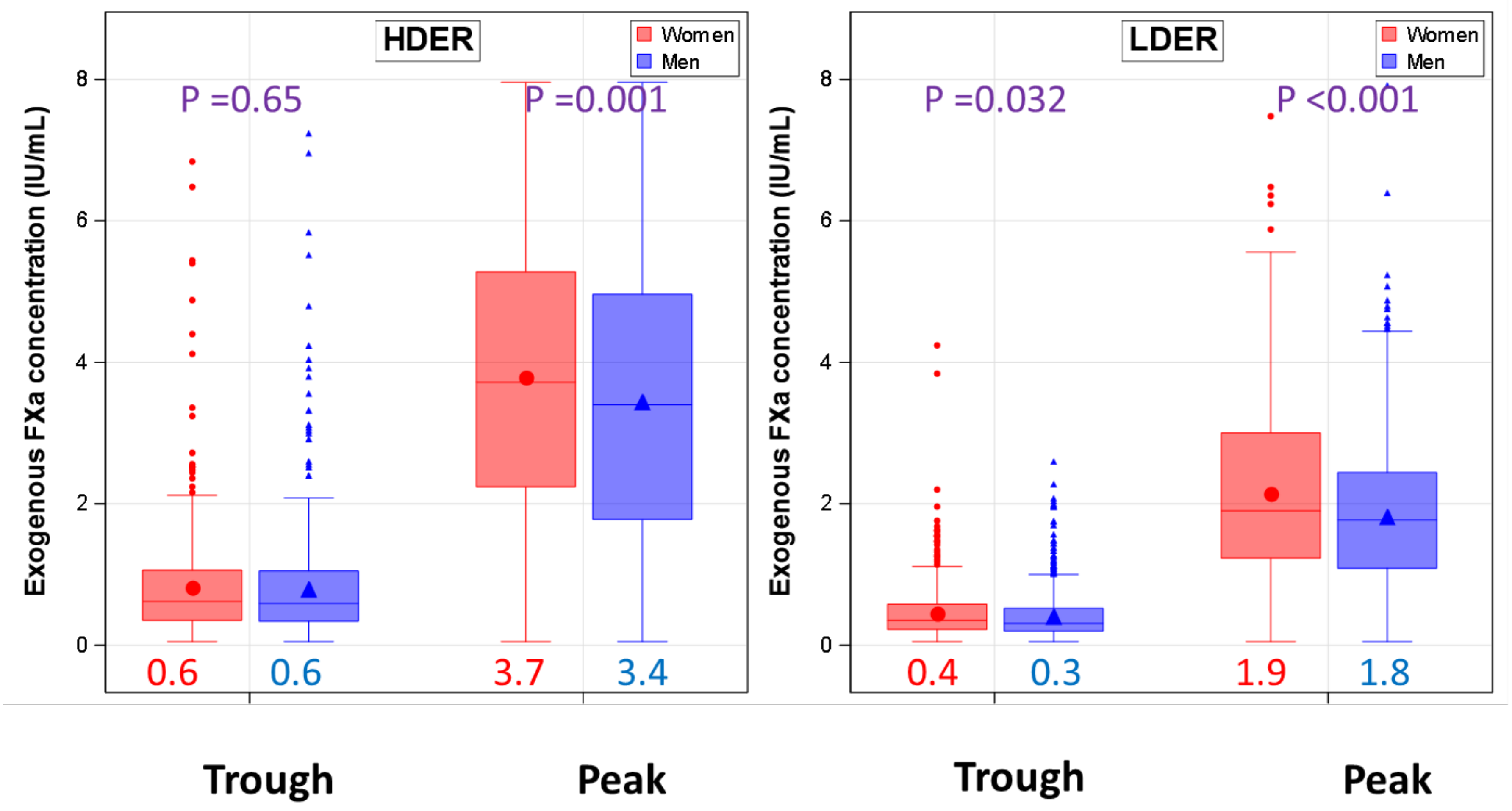

Supplemental Figure V: Exogenous Factor Xa concentrations with higher-dose (HDER) and lower-dose edoxaban regimen (LDER) according to dose-reduction (DR) criteria.

The difference between men and women was tested using the non-parametric Wilcoxon rank-sum test.

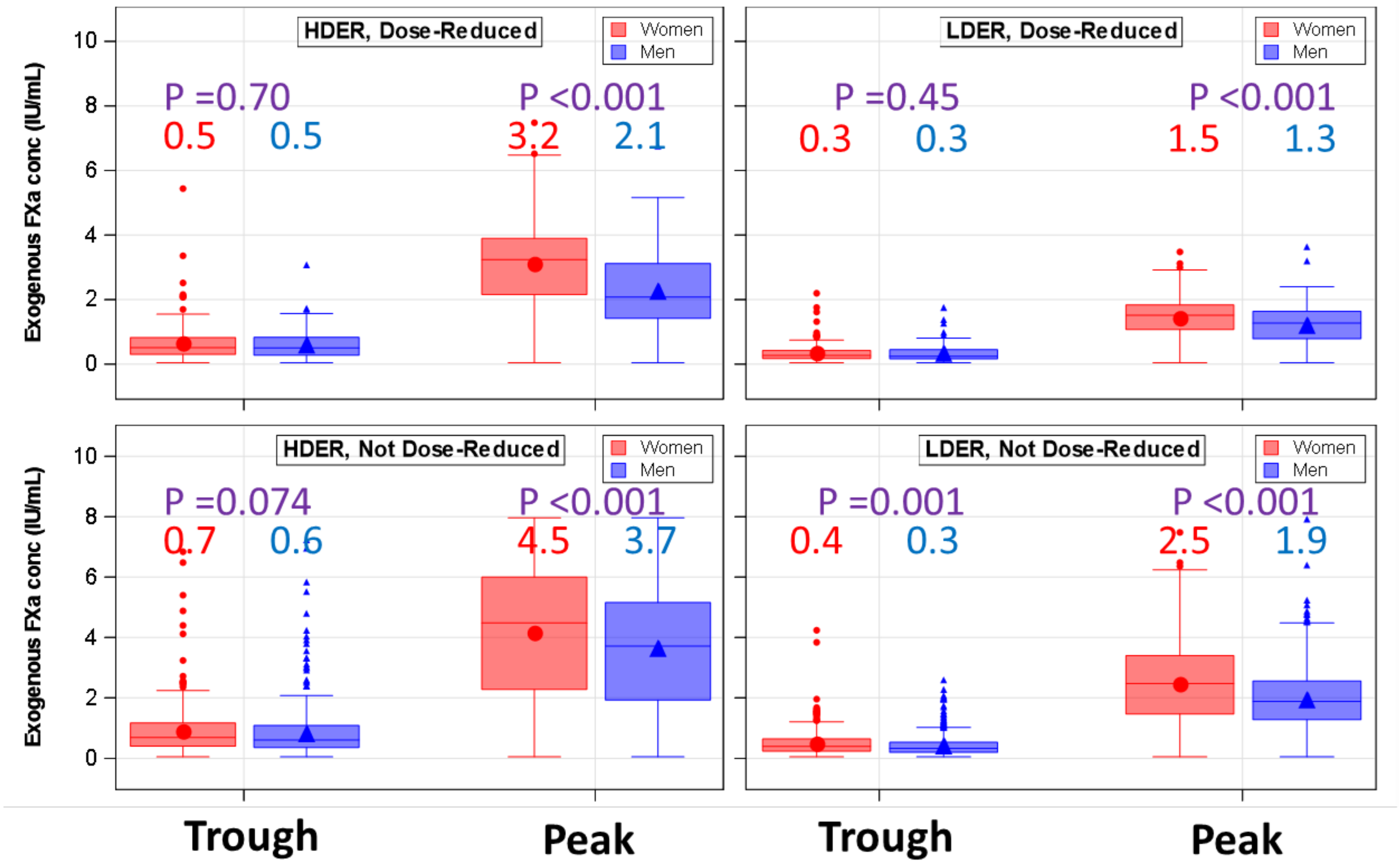

Supplemental Figure VI: Efficacy and safety of edoxaban versus warfarin stratified by sex using multivariable adjusted* Cox model. (The x-axis of the forest plot represents hazard ratio.)

*Adjusted for age, body mass index, race, smoking, history of stroke or TIA, diabetes, heart failure, hypertension, coronary artery disease, peripheral artery disease, dyslipidemia, hepatic disease, non-ICH bleed, AF type

	Sex	HDER Adj. HR (95% CI)	HDER x Sex Adj. P for interaction	LDER Adj. HR (95% CI)	LDER x Sex Adj. P for interaction
**Primary Endpoint**					
Stroke/SEE			0.99		0.77
	Female	0.86 (0.68-1.10)		1.16 (0.93-1.45)	
	Male	0.86 (0.70-1.06)		1.11 (0.92-1.35)	
**Secondary Endpoints**					
Stroke			0.89		0.73
	Female	0.88 (0.69-1.13)		1.17 (0.93-1.47)	
	Male	0.86 (0.70-1.06)		1.10 (0.90-1.35)	
Primary Hemorrhagic Stroke			0.038		0.31
	Female	0.30 (0.15-0.59)		0.24 (0.11-0.49)	
	Male	0.70 (0.46-1.06)		0.38 (0.23-0.63)	
Primary Ischemic Stroke			0.48		0.70
	Female	1.06 (0.81-1.39)		1.47 (1.14-1.89)	
	Male	0.93 (0.73-1.19)		1.38 (1.10-1.72)	
Myocardial infarction			0.76		0.97
	Female	1.02 (0.67-1.55)		1.22 (0.81-1.83)	
	Male	0.94 (0.70-1.25)		1.21 (0.92-1.58)	
Net clinical outcome			0.75		0.81
	Female	0.87 (0.77-0.99)		0.85 (0.75-0.96)	
	Male	0.90 (0.82-0.98)		0.83 (0.76-0.91)	
**Death**					
All-cause death			0.88		0.72
	Female	0.90 (0.76-1.07)		0.90 (0.76-1.07)	
	Male	0.92 (0.81-1.03)		0.87 (0.77-0.98)	
CV death			0.88		0.39
	Female	0.85 (0.69-1.04)		0.92 (0.76-1.13)	
	Male	0.87 (0.75-1.00)		0.83 (0.72-0.96)	
**Safety Endpoints**					
Major bleeding			0.30		0.83
	Female	0.72 (0.58-0.90)		0.46 (0.36-0.59)	
	Male	0.84 (0.71-0.98)		0.47 (0.39-0.57)	
Major and CRNM bleeding			0.007		0.106
	Female	0.75 (0.66-0.84)		0.56 (0.50-0.63)	
	Male	0.91 (0.83-0.99)		0.63 (0.58-0.70)	
Intracranial hemorrhage			0.004		0.64
	Female	0.20 (0.10-0.40)		0.28 (0.15-0.49)	
	Male	0.63 (0.44-0.89)		0.33 (0.21-0.51)	
Life-threatening or Fatal			<0.001		0.35
	Female	0.23 (0.13-0.39)		0.27 (0.17-0.44)	
	Male	0.71 (0.53-0.95)		0.36 (0.25-0.52)	
Any bleeding			0.004		0.12
	Female	0.76 (0.68-0.84)		0.60 (0.54-0.67)	
	Male	0.92 (0.85-0.99)		0.67 (0.62-0.73)	
Major GI bleeding			0.69		0.52
	Female	1.29 (0.92-1.81)		0.73 (0.50-1.07)	
	Male	1.19 (0.94-1.51)		0.63 (0.47-0.83)	

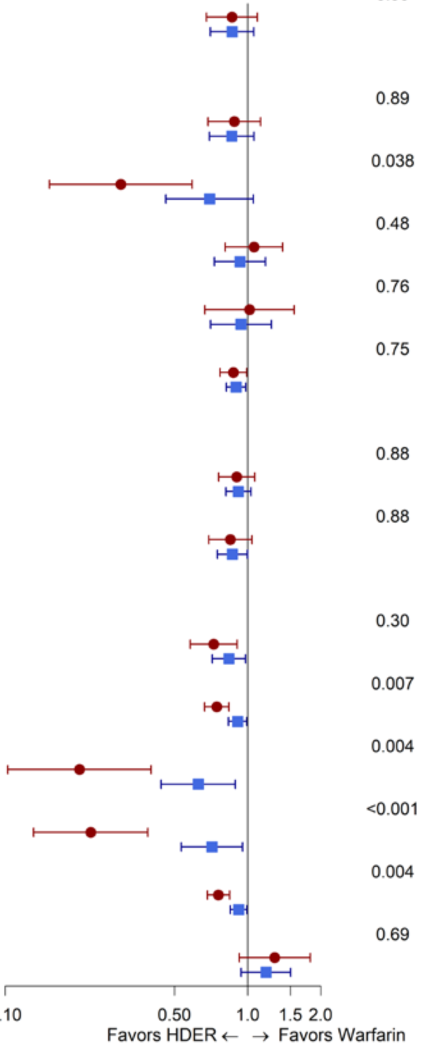

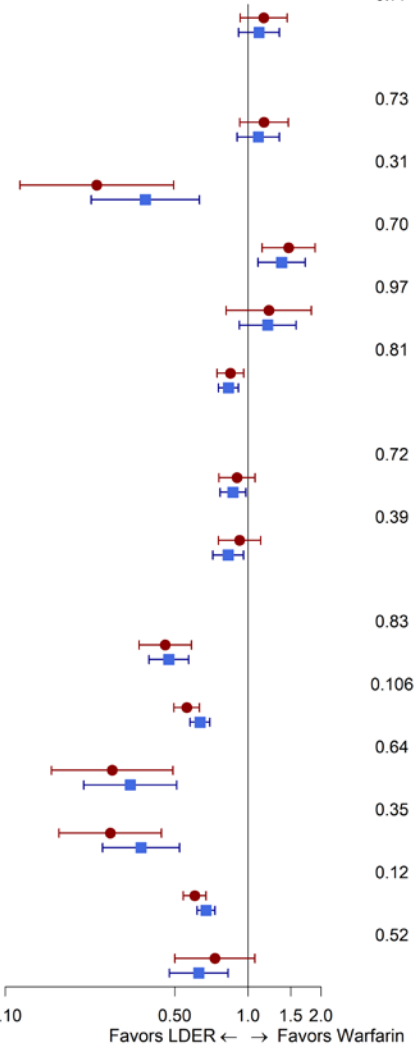

Supplemental Figure VII: Efficacy and safety of higher dose edoxaban regimen (HDER) and lower dose edoxaban regimen (LDER) versus warfarin in patients who met criteria for dose reduction stratified by sex (i.e., HDER 30 mg, LDER 15 mg). (The x-axis of the forest plot represents hazard

	Sex	N	Warfarin Annualized Event rate	HDER Annualized Event rate	LDER Annualized Event rate	HDER x Sex P for interaction	LDER x Sex P for interaction
**Primary Endpoint**							
Stroke/SEE						0.54	0.92
	Female	2928	2.8	2.2	3.2		
	Male	2428	2.5	2.4	3.0		
**Secondary Endpoints**							
Any stroke						0.91	0.95
	Female	2928	2.5	2.1	2.9		
	Male	2428	2.4	2.1	2.9		
Hemorrhagic stroke						0.048	0.17
	Female	2928	0.7	0.2	0.0		
	Male	2428	0.5	0.6	0.1		
Ischemic stroke						0.57	0.87
	Female	2928	1.8	1.9	2.9		
	Male	2428	1.8	1.6	2.8		
Myocardial infarction						0.48	0.86
	Female	2928	0.7	0.8	1.0		
	Male	2428	0.9	1.3	1.1		
**Death**							
All-cause death						0.98	0.91
	Female	2928	6.2	5.2	5.1		
	Male	2428	9.3	7.8	7.9		
CV death						0.78	0.75
	Female	2928	4.4	3.7	3.7		
	Male	2428	6.6	5.7	5.8		
**Safety Endpoints**							
Major bleeding						0.21	0.36
	Female	2911	4.7	2.5	1.3		
	Male	2419	5.0	3.6	1.8		
Major and CRNM bleeding						0.023	0.32
	Female	2911	17.6	10.2	7.9		
	Male	2419	18.8	14.8	9.7		
Intracranial hemorrhage						0.022	0.44
	Female	2911	1.3	0.3	0.1		
	Male	2419	1.2	0.9	0.2		
Life threatening or Fatal						0.026	0.20
	Female	2911	1.8	0.4	0.2		
	Male	2419	1.6	1.2	0.4		
Any bleeding						0.018	0.33
	Female	2911	22.1	13.4	11.2		
	Male	2419	22.3	18.1	12.8		
Major GI bleed						0.60	0.96
	Female	2911	1.3	1.2	0.8		
	Male	2419	1.6	1.7	0.9		

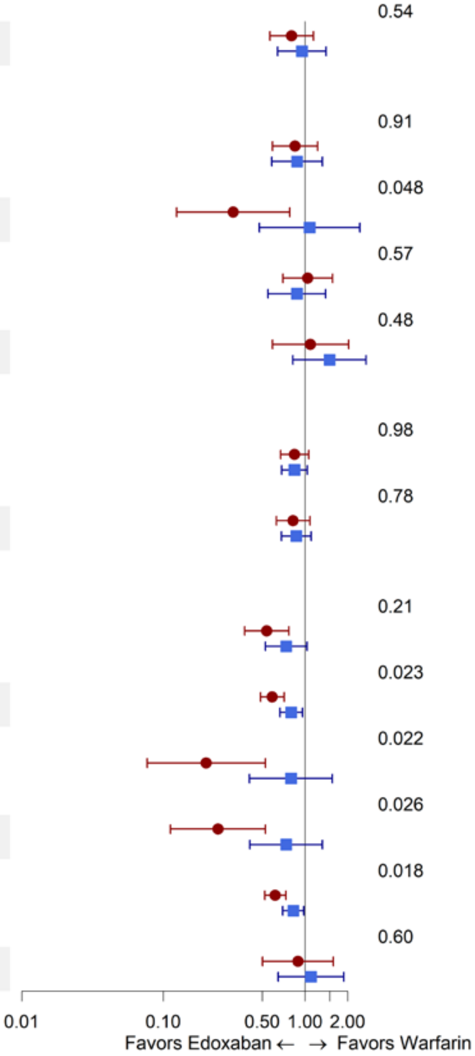

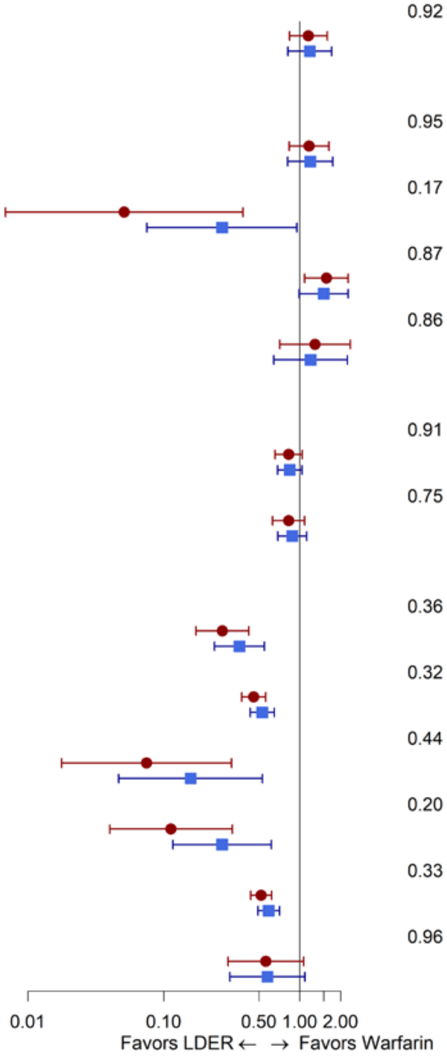

Supplemental Figure VIII: Efficacy and safety of higher dose edoxaban regimen (HDER) and lower dose edoxaban regimen (LDER) in patients who did not meet criteria for dose-reduction versus warfarin stratified by sex (i.e., HDER 60 mg, LDER 30 mg). (The x-axis of the forest plot represents hazard ratio.)

	Sex	N	Warfarin Annualized Event rate	HDER Annualized Event rate	LDER Annualized Event rate	HDER x Sex P for interaction	LDER x Sex P for interaction
**Primary Endpoint**							
Stroke/SEE						0.58	0.72
	Female	5112	1.6	1.5	1.8		
	Male	10637	1.5	1.3	1.6		
**Secondary Endpoints**							
Any stroke						0.68	0.70
	Female	5112	1.5	1.4	1.8		
	Male	10637	1.4	1.2	1.5		
Hemorrhagic stroke						0.20	0.67
	Female	5112	0.4	0.1	0.2		
	Male	10637	0.4	0.3	0.2		
Ischemic stroke						0.53	0.92
	Female	5112	1.2	1.3	1.6		
	Male	10637	1.0	1.0	1.3		
Myocardial infarction						0.64	0.67
	Female	5112	0.5	0.5	0.6		
	Male	10637	0.8	0.6	1.0		
**Death**							
All-cause death						0.52	0.40
	Female	5112	2.4	2.5	2.4		
	Male	10637	3.7	3.5	3.3		
CV death						0.67	0.14
	Female	5112	1.8	1.7	1.9		
	Male	10637	2.8	2.4	2.2		
**Safety Endpoints**							
Major bleeding						0.83	0.35
	Female	5095	2.7	2.5	1.7		
	Male	10601	3.2	2.8	1.6		
Major and CRNM bleeding						0.44	0.49
	Female	5095	11.6	10.4	7.5		
	Male	10601	11.6	10.9	7.9		
Intracranial hemorrhage						0.045	0.62
	Female	5095	0.7	0.1	0.3		
	Male	10601	0.7	0.4	0.3		
Life threatening or Fatal						0.005	0.93
	Female	5095	1.0	0.2	0.4		
	Male	10601	1.0	0.7	0.4		
Any bleeding						0.46	0.58
	Female	5095	14.8	13.3	10.0		
	Male	10601	14.8	14.0	10.5		
Major GI bleed						0.23	0.34
	Female	5095	0.9	1.6	0.8		
	Male	10601	1.3	1.5	0.8		

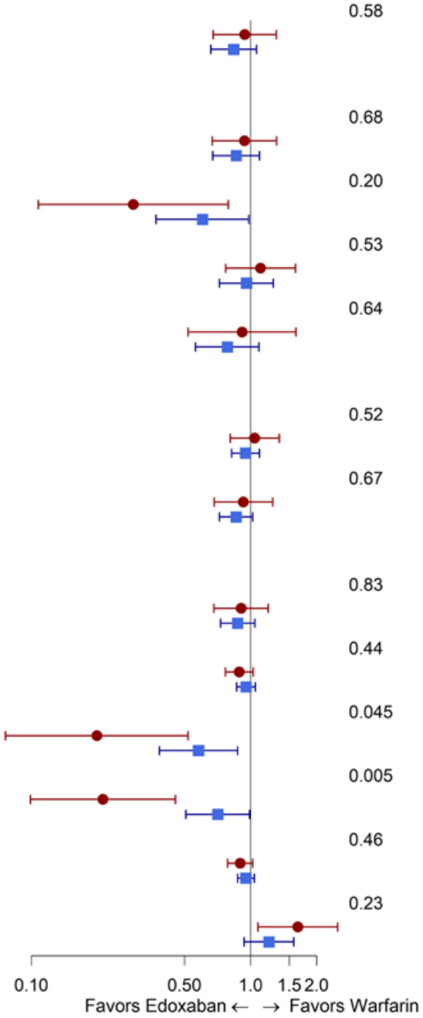

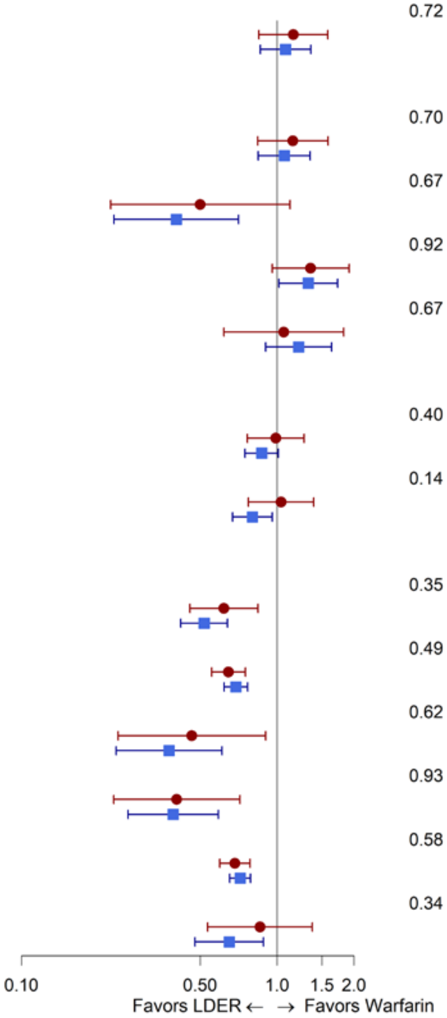

Supplemental Figure IX: Edoxaban versus warfarin on stroke and systemic embolic events in women by major subgroups. (The x-axis of the forest plot represents hazard ratio.)

		Total N	Warfarin Annualized Event rate	HDER Annualized Event rate	LDER Annualized Event rate	HDER x Subgrp P for interaction	LDER x Sex P for interaction
Age (yrs)							
	<65	1510	1.1	0.9	1.6	REF	REF
	65-<75	2753	1.8	1.5	2.1	1.00	0.48
	>=75	3777	2.5	2.3	2.8	0.86	0.44
Weight (kg)							
	<74	3914	2.5	2.2	2.9	REF	REF
	>=74	4126	1.5	1.3	1.8	0.99	0.80
CHADS2							
	<=3	6052	1.5	1.4	2.0	REF	REF
	>3	1988	3.5	2.8	3.4	0.53	0.23
Region							
	N. America	1598	1.8	1.9	2.1	REF	REF
	S. America	1081	2.2	1.8	2.3	0.61	0.72
	W. Europe	1233	1.8	2.3	2.1	0.47	0.93
	E. Europe	3008	1.7	1.5	2.6	0.70	0.47
	Asia	1120	3.1	1.6	2.3	0.15	0.24
Diabetes							
	No	5258	2.2	1.8	2.4	REF	REF
	Yes	2782	1.5	1.7	2.2	0.23	0.24
Heart Failure							
	No	3464	2.0	1.9	2.4	REF	REF
	Yes	4576	2.0	1.7	2.3	0.51	0.71
Stroke/TIA							
	No	5761	1.5	1.4	1.9	REF	REF
	Yes	2279	3.4	2.8	3.5	0.62	0.37
AF type							
	Paroxysmal	2432	0.9	2.0	1.9	REF	REF
	Persistent	1825	2.4	1.4	1.7	0.001	0.007
	Permanent	3780	2.5	1.8	2.9	0.001	0.109
Hypertension							
	No	409	2.2	2.3	4.4	REF	REF
	Yes	7631	2.0	1.7	2.2	0.60	0.11
Prior MI							
	No	7457	1.9	1.7	2.2	REF	REF
	Yes	582	3.1	2.0	3.1	0.42	0.81
Dose reduced							
	No	5112	1.6	1.5	1.8	REF	REF
	Yes	2928	2.8	2.2	3.2	0.52	1.00
Aspirin use							
	No	5903	1.9	1.8	2.3	REF	REF
	Yes	2135	2.4	1.6	2.3	0.12	0.31
Amiodarone use							
	No	7073	2.0	1.8	2.4	REF	REF
	Yes	967	1.7	1.2	1.6	0.55	0.47

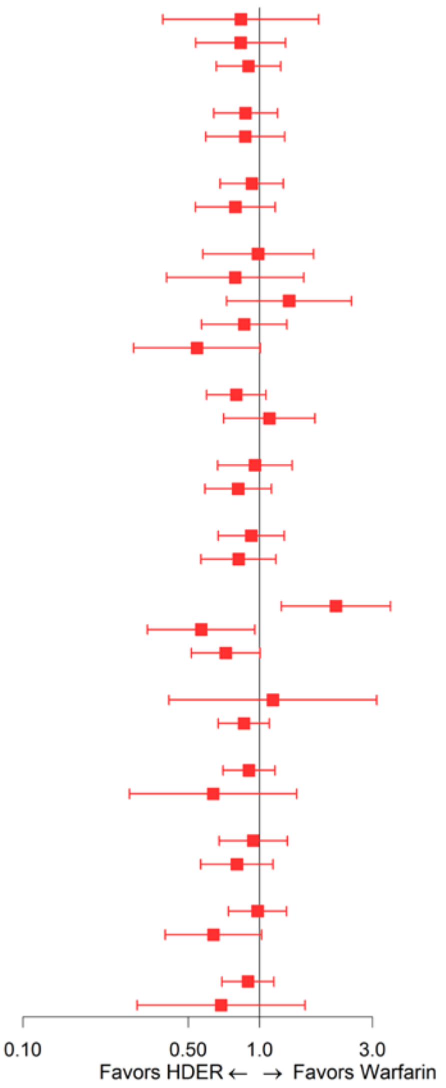

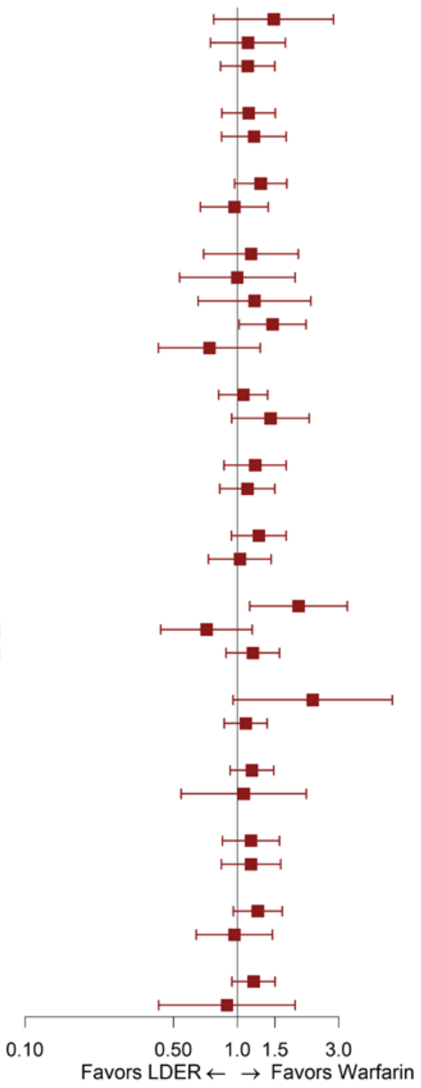

Supplemental Figure X: Edoxaban versus warfarin on major bleeding in women by major subgroups. (The x-axis of the forest plot represents hazard ratio.)

Subgroup		Total N	Warfarin Annualized Event rate	HDER Annualized Event rate	LDER Annualized Event rate	HDER x Subgrp P for interaction	LDER x Sex P for interaction
Age (yrs)							
	<65	1506	1.7	1.6	0.5	REF	REF
	65-<75	2745	2.8	2.2	1.4	0.64	0.28
	>=75	3755	4.7	3.2	2.1	0.40	0.36
Weight (kg)							
	<74	3890	3.9	2.6	1.5	REF	REF
	>=74	4116	2.9	2.3	1.6	0.43	0.20
CHADS2							
	<=3	6022	3.1	2.3	1.5	REF	REF
	>3	1984	4.2	3.2	1.7	0.89	0.60
Region							
	N. America	1591	4.4	4.4	2.8	REF	REF
	S. America	1078	3.7	2.6	1.3	0.37	0.22
	W. Europe	1227	4.0	2.7	1.4	0.32	0.18
	E. Europe	2997	2.3	1.3	1.0	0.090	0.31
	Asia	1113	4.3	3.0	1.6	0.34	0.21
Diabetes							
	No	5232	3.2	2.4	1.4	REF	REF
	Yes	2774	3.6	2.5	1.8	0.69	0.58
CHF							
	No	3442	3.7	2.9	1.6	REF	REF
	Yes	4564	3.1	2.2	1.5	0.75	0.56
Stroke/TIA							
	No	5733	3.3	2.3	1.6	REF	REF
	Yes	2273	3.4	3.0	1.3	0.36	0.38
AF type							
	Paroxysmal	2421	3.0	3.4	1.1	REF	REF
	Persistent	1815	3.9	1.5	1.5	0.002	0.74
	Permanent	3770	3.3	2.3	1.9	0.072	0.12
Hypertension							
	No	407	4.1	2.9	1.5	REF	REF
	Yes	7599	3.3	2.5	1.5	0.96	0.76
Prior MI							
	No	7424	3.5	2.5	1.5	REF	REF
	Yes	581	1.8	2.9	1.8	0.11	0.11
Dose reduced							
	No	5095	2.7	2.5	1.7	REF	REF
	Yes	2911	4.7	2.5	1.3	0.028	0.003
Aspirin use							
	No	5885	3.0	2.4	1.3	REF	REF
	Yes	2120	4.5	2.8	2.3	0.32	0.55
Amiodarone use							
	No	7042	3.4	2.5	1.6	REF	REF
	Yes	964	2.6	2.5	0.8	0.41	0.42

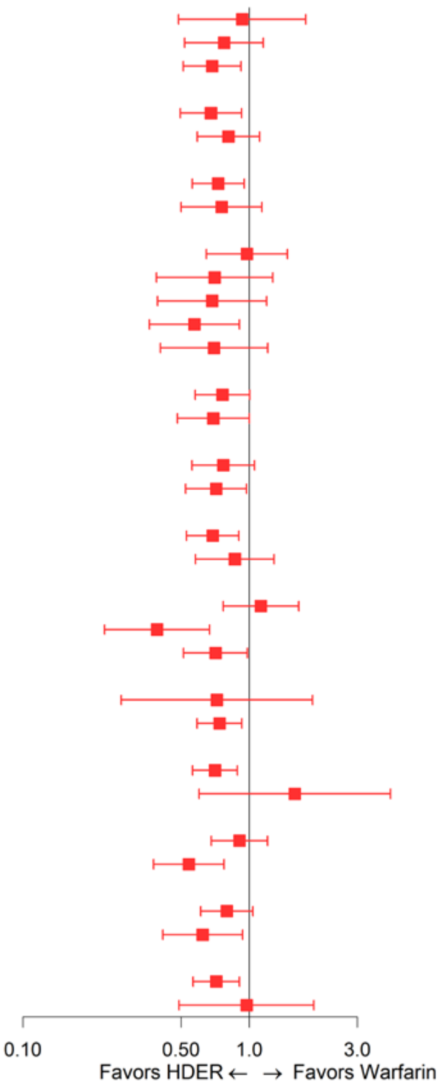

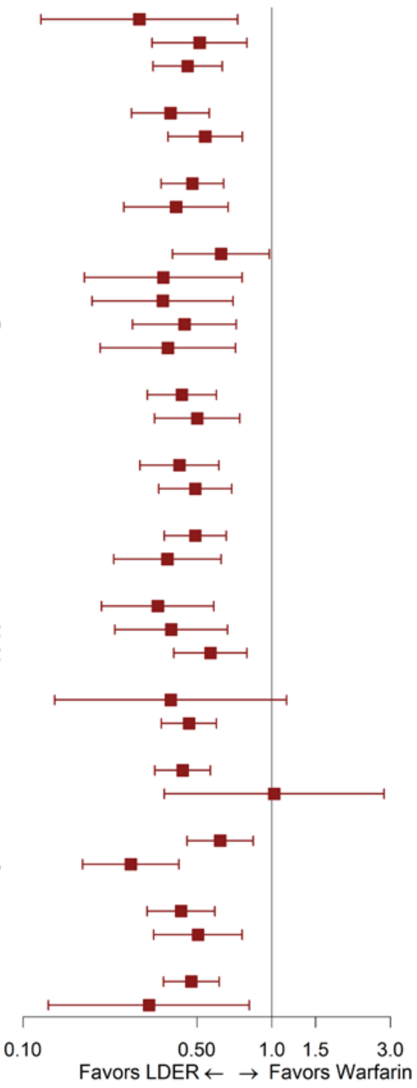

Supplement: Supplementary file 1 [file cir-143-673-s001.pdf]
